# ECM-dependent regulation of septin 7 in focal adhesions promotes mechanosensing and functional response in fibroblasts

**DOI:** 10.1016/j.isci.2024.111355

**Published:** 2024-11-09

**Authors:** Wesley Sturgess, Swathi Packirisamy, Rodina Geneidy, Pontus Nordenfelt, Vinay Swaminathan

**Affiliations:** 1 Division of Oncology, Department of Clinical Sciences, Lund University, Lund, Sweden; 2 Wallenberg Centre for Molecular Medicine, Lund University, Lund, Sweden; 3 Division of Infection Medicine, Department of Clinical Sciences, Lund University, Lund, Sweden; 4 Department of Laboratory Medicine, Clinical Microbiology, Skåne University Hospital Lund, Lund Universty, Lund, Sweden

**Keywords:** Mechanobiology, Cell biology, Stem cells research

## Abstract

Fibroblasts are adherent cells that maintain tissue homeostasis by sensing and responding to the extracellular matrix (ECM). Focal adhesions (FAs) link these ECM changes to actomyosin dynamics through changes in its composition, influencing cellular response. Septin-7 (Sept-7) has previously been found in FA proteomics studies and shown to influence ECM sensing. Using total internal reflection microscopy, we found that ECM-mediated integrin activation regulates spatially distinct Sept-7 structures in FAs. In perinuclear regions, ECM binding stabilized Sept-7 bundles at the back of FAs, while in the core of peripheral FAs high integrin activation promoted elongation of Sept-7 structures. Ventral Sept-7 structures were crucial for ECM sensing, impacting region-specific FA elongation, stabilization, and contributing to fibroblast mechanosensitivity. Taken together, our results suggest that ECM and integrin-dependent regulation of ventral Sept-7 structures plays a pivotal role in fibroblast ECM sensing and mechanotransduction through its recruitment and assembly into FA subpopulations.

## Highlights

• Sept-7 localizes to FAs in a spatially (peripheral vs. perinuclear) distinct manner
• Sept-7 localization to FAs is dependent on ECM binding and integrin activation
• Sept-7 promotes elongation of peripheral FAs and stabilization of perinuclear FAs
• Sept-7 increases cellular sensitivity to changes in physical cues from the ECM

## Graphical abstract

**Figure:**
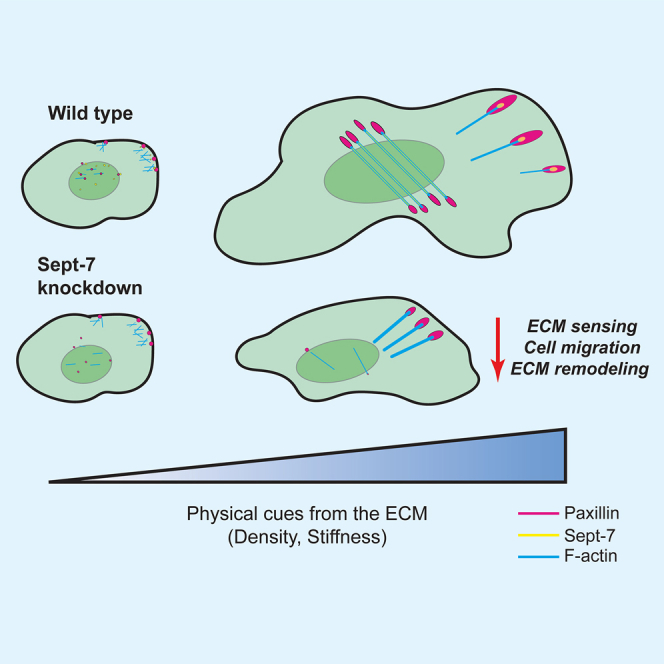

## Introduction

The extracellular matrix (ECM) is a complex, multicomponent, and dynamically changing non-cellular structure surrounding most cell types and tissues in our body.^1^^,^^2^^,^^3^ The proper regulation of the ECM’s physical and biochemical properties is critical for several cellular processes, from cell specification and organogenesis to wound healing and immune responses and is altered in diseases such as cancer and atherosclerosis.^3^^,^^4^^,^^5^^,^^6^^,^^7^^,^^8^ These key physical and biochemical properties of the ECM not only include its stiffness and architecture but also the specific ECM ligand composition and its local density. For example, it has been shown *in vivo* and *in vitro* that several cell types including breast cancer cells and immune cells can migrate on density gradients of ECM ligands such as fibronectin (FN) or ECM immobilized density gradients of chemokines (CCL21).^9^^,^^10^^,^^11^ Similarly, it is also known that the local ECM density and composition surrounding both normal tissue, such as the mammary gland, and cancerous tissue can have a significant impact on tissue remodeling as well as on tumor cell growth and proliferation.^12^^,^^13^ This suggests that cells in our body have highly intricate mechanisms to sense ECM stiffness, composition, density and architecture and regulate these properties. Fibroblasts are one of the primary cell types responsible for this regulatory role and do so by sensing changes in ECM properties and responding to these changes by producing, modifying, and remodeling the ECM.^14^ Fibroblasts thus need to have highly sensitive ECM-sensing mechanisms that allow it to sense small and distinct changes in the ECM environment that downstream trigger highly specific cellular responses. This important function is primarily mediated by integrin-based focal adhesions (FAs).^15^^,^^16^^,^^17^^,^^18^

FAs comprise of the integrin family of receptors that indirectly couples the ECM to the actin and microtubule cytoskeleton via a network of proteins termed the adhesome. The complex regulation of recruitment and activity of adhesome proteins coupled to ECM-dependent integrin activation modulates the biophysical and biochemical coupling between the ECM and the cytoskeleton and drives the sensing of different ECM cues as well as regulation of downstream cellular responses.^19^ While we now know of several mechanisms by which FAs can sense changes in ECM stiffness and architecture, mechanisms that fine-tune the sensitivity and specificity of ECM sensing and cellular response is still not completely known. Proteomics using different FA isolation techniques and across several cell types have identified more than 2000 proteins in the adhesome,^20^^,^^21^^,^^22^ and this has led to the hypothesis that while key FA proteins such as integrins and talin are essential for cells to adhere to the ECM and trigger mechanotransduction cascades, several of the other FA-associated proteins could be more responsible for promoting sensitivity and specificity to changes in the ECM.^23^^,^^24^ Currently however, several of these proteins have no directly identified function in regulating FAs or in sensing specific changes in the mechanical ECM. Additionally, it has also been hypothesized that heterogeneity in FA subpopulations within a cell can promote ECM sensitivity and specificity through local changes in composition or dynamics.^25^^,^^26^ However, since most of the proteomics studies rely on bulk isolation of FAs in the cell, information about composition of FA subpopulations within a cell based on location, maturation level or other subcellular states and whether these subpopulations play specific roles is not known.

To address these specific questions, we focused on investigating the role of Sept-7 which is one of the most enriched septins in the adhesome.^22^ Septins are GTP-binding proteins that self-assemble into oligomers and polymers and form higher ordered structures either with linear or with curved filaments and rings.^27^ Several studies have identified important roles for septins in regulating FA properties including its formation and maturation rate as well as its disassembly.^28^^,^^29^^,^^30^^,^^31^^,^^32^ In addition, Sept-7 containing bundles are found in proximity to FAs at the cell periphery^28^ as well as in the perinuclear area along actin fibers where through interactions with F-actin, Sept-7 is thought to play an important role in sensing ECM stiffness.^33^ However, in spite of Sept-7 being found in FA proteomic studies and evidence suggesting interactions between Sept-7 and other FA proteins, septins, and specifically Sept-7 is considered to be excluded from FAs and its direct relationship with changing ECM cues and integrin activation is not known.^34^^,^^35^^,^^36^ We aimed to resolve these differences and understand the relationship between ECM sensing, integrin activation and of Sept-7 in FAs in this study. By using total internal reflection microscopy (TIRFM) to image the cell-ECM interface with high resolution, we found that Sept-7 forms distinct structures on the ventral surface of cells that localize to and in proximity of FAs of mouse embryonic fibroblasts (MEFs). In addition to location specific architecture, Sept-7 localization was also spatially and temporally distinctly localized within FA subpopulations. Sept-7 recruitment to the back of perinuclear FAs (in close proximity to the nucleus) and formation of higher order bundles was dependent on binding to the ECM protein fibronectin (FN) while FN binding and high integrin activation was required to form elongated FA-like Sept-7 structures that co-localized within the core of peripheral FAs (closer to the leading edge). To test the function of Sept-7 in FA regulation, we downregulated Sept-7 expression in MEFs and found that this led to a dramatic loss in the perinuclear FA population by affecting perinuclear FA maturation rate and lifetime, with only a minor effect on peripheral FA elongation. However, both of these distinct populations contributed differentially to the overall sensitivity of MEFs to changes in ECM density and integrin activation. In addition, we also found that ventral Sept-7 promoted the ability of MEFs to remodel and clear the ECM. Taken together, our work identifies Sept-7 as an FA and FA-associated protein that via ECM and integrin activation dependent changes in its architecture and localization regulates FA populations. Through these mechanisms, Sept-7 promotes the sensitivity of fibroblasts to regulate the physical properties of the ECM.

## Results

### Sept-7 localizes in spatially and temporally distinct patterns in FA sub-populations

**Figure 1:**
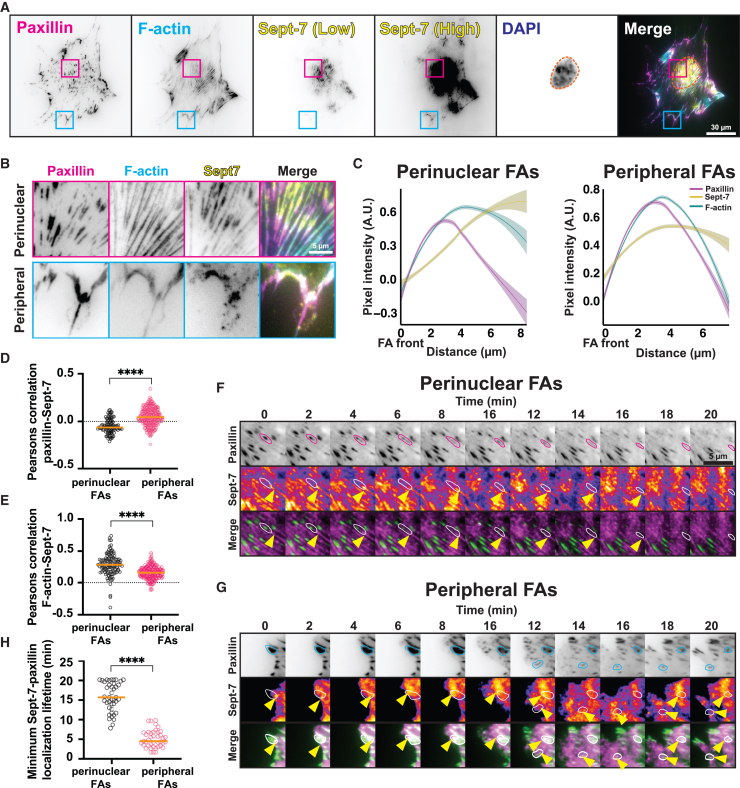
Sept-7 localizes in spatially and temporally distinct patterns in FA sub-populations (A) Representative TIRFM images of MEFs stained with paxillin (magenta), F-actin (cyan), and Sept-7, (yellow, low, and high contrast) of cells plated on 10 μg/mL FN. Magenta and cyan boxes highlight perinuclear and peripheral FAs respectively; scale bar, 30 μm. (B) Insets of perinuclear (magenta), and peripheral (cyan) FAs from (A); scale bar, 5 μm. (C) Curve plots showing normalized line scan intensities of paxillin (magenta), Sept-7 (yellow), and F-actin (cyan), for perinuclear (left) and peripheral (right) FAs, shaded curves represent SD, *n* = 3 replicates, 38–43 line scans. (D and E) Quantification of colocalization between paxillin and Sept-7, and F-actin and Sept-7 respectively, for perinuclear and peripheral FAs, *n* = 3 replicates, 1050–2270 FAs. (F and G) Montages taken from live TIRFM movie of perinuclear and peripheral FAs from MEFs co-transfected with paxillin-mCherry (gray, top) and Sept-7-YFP (fire, middle), and merged channels (bottom). Selected FAs are circled, and colocalized Sept-7 indicated with yellow arrows; scale bar, 5 μm. (H) Quantification of the minimum Sept-7-paxillin localized lifetime, *n* = 3 replicates, 32–40 FAs. Statistical analysis was performed using Mann-Whitney test, ∗∗∗∗*p* < 0.0001, orange horizonal lines show medians.

Based on microscopy-based localization data, Sept-7 is thought to be excluded from FAs which contradicts proteomics studies where Sept-7 is found to be enriched in FAs.^22^^,^^37^^,^^38^ Since FA composition and function is highly dependent on the cell type, we sought to clarify these differences using fibroblasts which are highly contractile adherent cells that form large, dynamic FAs for motility and ECM sensing. We plated MEFs on glass-bottom dishes coated with 10 μg/mL fibronectin (FN) which allows for formation of robust integrin-dependent FAs that promote optimal ECM sensing for cell migration and mechanotransduction.^39^ Cells were then fixed and immuno-stained for the FA protein paxillin, F-actin, and Sept-7 and imaged using TIRFM (Figures 1A and 1B). Consistent with previous results, we found filamentous Sept-7 decorating ventral actin stress fibers near paxillin enriched FAs beneath and around the nucleus (Figures 1A and 1B-top panel).^27^^,^^32^^,^^34^^,^^40^^,^^41^^,^^42^ In addition, we also observed Sept-7 near the front of the cell in the lamellipodia as well as in elongated punctate structures in the lamella of the cell where it seemed to co-localize with the paxillin signal (Figure 1B, lower panel). The lamellipodia and lamella-localized Sept-7 signal while robust however was relatively weak compared to the signal from the filamentous structures found in proximity of the nucleus. Due to the 2 distinct structures of Sept-7 observed in the proximity of FAs, and to determine the exact location of Sept-7 relative to F-actin and paxillin, we first classified the paxillin stained FAs as either perinuclear (connected to ventral actin stress fibers beneath the nucleus) or peripheral (located closer to the edge of the cell). We then generated a series of line scans across the FAs and plotted the average location of Sept-7 and F-actin relative to the location of paxillin within the FA (Figure 1C). This analysis showed that in perinuclear FAs, filamentous Sept-7 localized toward the rear of the FA with its average intensity peak outside of the FA with very little overlap with paxillin. In contrast, at the peripheral FAs, Sept-7 punctate structures peaked more centrally to both the paxillin and the F-actin peak and terminated at the end of the FA (Figure 1C). To further confirm these distinct localization patterns, we performed a pixel-by-pixel Pearson’s correlation analysis between paxillin, F-actin and Sept-7 in the 2 FA populations and found that there was a positive correlation between paxillin and Sept-7 as well as between F-actin and Sept-7 in the peripheral FAs, while there was no correlation between paxillin and Sept-7 in perinuclear FAs (Figures 1D and 1E). In contrast to paxillin, F-actin and Sept-7 showed a high positive correlation in the perinuclear FAs (Figure 1E). To test if Sept-7 associated peripheral and perinuclear FAs were compositionally unique within the cell, we immuno-stained Sept-7 with vinculin, FAK and active α5β1 integrins (Figure S1A). While we couldn’t find compositionally distinct FAs with these proteins, Pearson’s correlation analysis showed that within peripheral FAs, Sept-7 only positively co-localized with paxillin and F-actin but not with either vinculin, FAK or active α5β1 (Figure S1C). Similarly, we found that in perinuclear FAs, Sept-7 only positively co-localized with F-actin and not with any other FA proteins (Figure S1B). The spatially distinct FA subpopulation-dependent location and structure of Sept-7 suggested to us that there were at least 2 separate populations of Sept-7 containing structures on the ventral-surface of the cell in and around FAs. We found a similar FA sub-population dependent localization pattern for Sept-9 which forms oligomeric complexes with Sept-7, suggesting that these structures could be oligomers of septins (Figure S2).

To investigate if the dynamics of Sept-7 in these sub-populations was also distinct, we co-expressed Sept-7-YFP with paxillin-mCherry in MEFs and imaged cells 4 h after plating them on 10 μg/mL FN coated glass-bottom dishes using TIRFM (Figures 1F and 1G, supplementary movies (Videos S1 and S2)). Time-lapse imaging confirmed distinct dynamics of localization at the peripheral FAs compared to perinuclear FAs. In perinuclear FAs, Sept-7 bundles localized to the back of the FA and remained stably associated during the entire lifetime of the perinuclear FA (Figure 1F) which lasted relatively long (at least >7 min) (Figure 1H). In contrast, Sept-7 puncta dynamically and transiently associated with the core of the peripheral FAs during the adhesion lifetime (Figure 1H). However, within the spatial and temporal resolution of our imaging, we found no specific correlation between the dynamics of the peripheral FAs and of the Sept-7 puncta (Figure 1G). Taken together, our data here shows that Sept-7 localizes to or in proximity of FAs of adherent cells in distinct structures with dynamics and FA localization dependent on the FA subpopulation.

Video S1. Perinuclear region of MEFs transfected with Sept-7 and paxillin, related to figure 1 TIRFM time-lapse imaging zoom in of perinuclear region of MEFs transfected with Sept-7-YFP (fire) and paxillin-mCherry (gray, inverted). Images taken every 10 s (elapsed time shown in min:s). Montages in Figure 1F correspond to this movie

Video S2. Peripheral region of MEFs transfected with Sept-7 and paxillin, related to figure 1 TIRFM time-lapse imaging zoom in peripheral region of MEFs transfected with Sept-7-YFP (fire) and paxillin-mCherry (gray, inverted). Images taken every 10 s (elapsed time shown in min:s). Montages in Figure 1G correspond to this movie

### ECM-mediated integrin activation differentially promotes formation of spatially distinct ventral Sept-7 structures and its association with FAs

**Figure 2:**
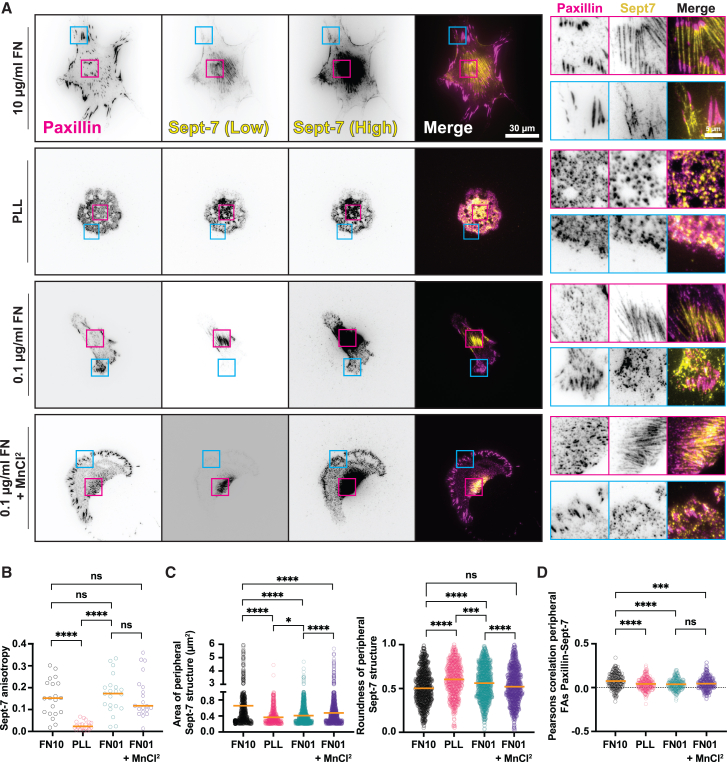
ECM-mediated integrin activation differentially promotes formation of spatially distinct ventral Sept-7 structures and promotes its association with FAs (A) TIRFM images showing paxillin (magenta), and Sept-7 (yellow, low, and high contrast), of MEFs plated on 10 μg/mL FN, PLL, 0.1 μg/mL FN, and 0.1 μg/ml FN + MnCl^2^, magenta and cyan insets highlight perinuclear and peripheral FAs respectively; scale bars, 30 μm (main images), 5 μm (insets). (B) Quantification of Sept-7 anisotropy of MEFs plated on 10 μg/mL FN, PLL, 0.1 μg/mL FN, and 0.1 μg/ml FN + MnCl^2^, *n* = 2 replicates, 20–22 cells. (C) Quantification of peripheral Sept-7 structure area (left plot), and roundness (right plot), for MEFs plated on 10 μg/mL FN, PLL, 0.1 μg/mL FN, and 0.1 μg/ml FN + MnCl^2^, *n* = 3 replicates, 627–1418 structures. (D) Quantification of colocalization between paxillin and Sept-7 at peripheral FAs of cells plated on 10 μg/mL FN, PLL, 0.1 μg/mL FN, and 0.1 μg/ml FN + MnCl^2^, *n* = 3 replicates, 2030–3400 FAs. All statistics performed using Kruskal-Wallis and Dunn’s multiple comparisons test, ∗∗∗∗*p* < 0.0001, ∗∗∗*p* < 0.001, ∗∗*p* < 0.01, ∗*p* < 0.05, ns, not significant, orange horizonal lines show medians.

Our data on Sept-7 localization and its dynamics in FAs suggests a mechanistic link between Sept-7 recruitment to FAs and ECM-mediated integrin activation which regulates the formation and fate of FAs. To investigate this, we plated MEFs on glass-bottom dishes coated with poly-L-lysine (PLL - to prevent ECM mediated integrin activation), or 0.1 μg/mL FN (to achieve low levels of integrin activation) and fixed and stained the cells for Sept-7 and paxillin to compare with cells on 10 μg/mL FN (Figure 2A). As expected, cells coated on PLL had no large perinuclear or peripheral FAs, shown by diffused paxillin staining, compared to cells on 10 μg/mL FN, which was further confirmed by quantification of FA size which showed an expected decrease in size (Figure S3A). The loss of FAs on cells plated on PLL also coincided with loss of filamentous Sept-7 structures in the perinuclear region with Sept-7 instead forming puncta or rings throughout the cell (Figure 2A). Increasing the FN concentration to 0.1 μg/mL FN resulted in robust formation of perinuclear Sept-7 bundles while peripheral Sept-7 structures though formed, were relatively small in the vicinity of FA structures unlike cells on 10 μg/mL FN (Figure 2A). Interestingly, the changes in perinuclear Sept-7 structures did not coincide with changes in FA size, since perinuclear and peripheral FA size were still small for cells on 0.1 μg/mL FN compared to 10 μg/mL FN (Figure S3A). To quantify the ECM-dependent morphologies of Sept-7 structures and its localization, we measured Sept-7 anisotropy for the perinuclear bundles and the size and shape of peripheral Sept-7 structures and its colocalization with paxillin in peripheral FAs across these conditions (Figures 2B–2D). This quantification showed a robust increase in perinuclear Sept-7 anisotropy upon ECM binding at 0.1 μg/mL FN compared to cells on PLL (Figure 2B). Peripheral Sept-7 structures on the other hand, only started getting fully elongated at the highest ECM density of 10 μg/mL FN with a small increase in size and elongation on 0.1 μg/mL FN compared to PLL (Figure 2C). Additionally, we found loss of colocalization between paxillin and Sept-7 pixels in peripheral FAs in cells plated on 0.1 μg/mL FN or PLL compared to cells on 10 μg/mL FN (Figure 2D). Taken together, this suggests that while low density ECM ligand binding is sufficient to induce complete and robust formation of perinuclear Sept7 bundles, it is not sufficient to localize and elongate Sept-7 structures in peripheral FAs which instead increases with increasing ECM density.

Based on the difference in Sept-7 localization in peripheral FAs between 0.1 μg/mL FN and 10 μg/mL FN, we then hypothesized that its peripheral localization is more sensitive to changes in integrin activation and thus requires higher integrin activation than that at 0.1 μg/mL FN. To test this, we pre-treated cells with 1 mM MnCl_2_ to shift surface expressed integrins to an extended (primed) conformation, and then plated them on 0.1 μg/mL FN prior to fixing and immunostaining for paxillin and Sept-7 (Figure 2A, bottom panel).^43^^,^^44^ Analysis of Sept-7 structures and its localization showed that while the increase in integrin activation did not significantly change perinuclear Sept-7 anisotropy compared to untreated cells on 0.1 μg/mL FN or its colocalization with paxillin, in peripheral regions it was sufficient to increase the size of Sept-7 structures and restore their elongation to levels of 10 μg/mL FN (Figures 2B–2D). To further test if the effect of increased integrin activation was dependent on FAK activation and thus integrin signaling, we plated cells on 10 μg/mL FN and inhibited FAK activity using the FAK inhibitor PF-573228 but found that it had a small but statistically insignificant effect on Sept-7 bundles in the perinuclear region and no effect on Sept-7 localization to peripheral FAs (Figures S3B–S3D). However, we found that inhibiting contractility using blebbistatin on MEFs plated on 10 μg/mL FN resulted in loss of perinuclear Sept-7 bundles, reduced its anisotropy and affected the elongation of peripheral Sept-7 structures (Figures S3E–S3G). Taken together, these results indicate that the spatially distinct ventral localization and morphology of Sept-7 depends on specific ECM properties with perinuclear Sept-7 bundles dependent on just ECM binding and actomyosin contractility while peripheral FA-localized elongated Sept-7 structures requires ECM-binding that triggers high levels of integrin activation and contractility.

### Sept-7 promotes the maturation and stabilization of perinuclear FAs

**Figure 3:**
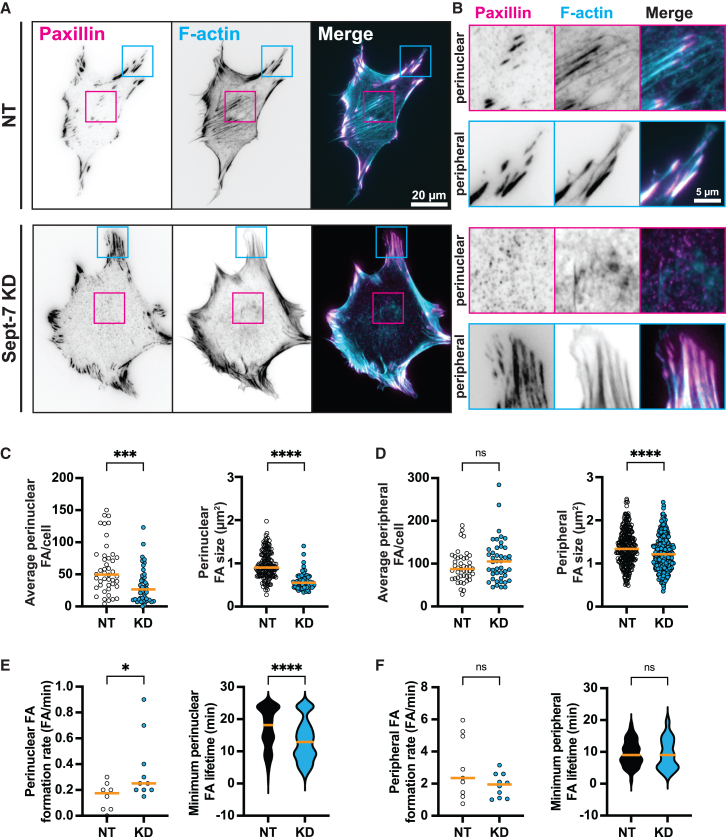
Sept-7 promotes the maturation and stabilization of perinuclear FAs (A) TIRFM images show MEFs transfected with NT (top panels) or Sept-7 KD (lower panels) siRNA and labeled with paxillin (magenta) and F-actin (cyan), magenta and cyan boxes highlight perinuclear and peripheral FAs respectively; scale bar, 20 μm. (B) ROIs of perinuclear (magenta) and peripheral (cyan) FAs from (A); scale bar, 5 μm. (C and D) Quantification of perinuclear (C) and peripheral (D) average FA number and FA size for NT and Sept-7 KD MEFs plated on 10 μg/mL FN, *n* = 3 replicates, 670–3160 FAs. (E and F) Quantification of perinuclear (E) and peripheral (F) FA formation rate and minimum FA lifetime for NT and Sept-7 KD MEFs plated on 10 μg/mL FN *n* = 2–3 replicates, 53–75 FAs. All statistics performed using Mann-Whitney test, ∗∗∗∗*p* < 0.0001, ∗∗∗*p* < 0.001, ∗∗*p* < 0.01, ∗*p* < 0.05, ns, not significant, orange horizonal lines show medians.

Due to the strong coupling between localization of Sept-7 structures in and around FAs and integrin activation, we next investigated the role of Sept-7 in FA formation and dynamics. To do this, we used pooled siRNAs to knockdown Sept-7 (Sept-7 KD) expression in MEFs (Figure S4A) and quantified FA morpho-dynamics using TIRFM. To first measure static FA properties, we fixed Sept-7 KD or non-targeting (NT) siRNA control cells plated on 10 μg/mL FN and stained for paxillin and F-actin (Figure 3A). Strikingly, we observed a near complete loss of large perinuclear FAs and associated ventral stress fibers in Sept-7 KD cells compared to NT controls, accompanied with smaller peripheral FAs (Figures 3A and 3B). Quantification of FA number and size confirmed our observations, showing a significant reduction in the number of perinuclear FAs in Sept-7 KD cells compared to NT control (Figure 3C). In addition to the reduced number, Sept-7 loss also resulted in the remaining perinuclear FAs to be significantly smaller compared to the controls (Figure 3C). While knocking down Sept-7 had no effect on the average number of peripheral FAs), quantification of FA size revealed a significant reduction in peripheral FA size compared to the NT control (Figure 3D). To test if these effects of Sept-7 loss was specific to the ventral surface of the cell, we used 3D Structured Illumination Microscopy (SIM) to image the ventral actin stress fibers which are linked to perinuclear FAs, and the apical perinuclear actin cap which traverse the cell and attach to peripheral FAs (Figure S4B, cartoon). In NT siRNA control cells, we again found robust actin stress fibers above and below the nucleus which were associated with Sept-7 bundles on the ventral side and smaller Sept-7 structures on the apical side (Figure S4B, left panel). Loss of Sept-7 however only affected the ventral actin stress fibers linked to perinuclear FAs resulting in loss of thick bundles with little or no effect on the apical perinuclear actin cap (Figure S4B, right panel). In addition, we also found that the perinuclear FAs lost due to loss of Sept-7 were not specific to paxillin positive FAs as we found significant size reductions for vinculin, FAK, active α5β1 integrins and tensin-1 labeled perinuclear FAs (Figure S4C). There was, however, no reduction in either number or size of these individually labeled peripheral FAs (Figure S4D).

Next, due to the strong effect on perinuclear FAs, we asked if the significant reduction in the number and size of perinuclear FAs accompanying Sept-7 loss was due to reduction in formation of new perinuclear FAs or due to changes in the perinuclear FA lifetime and growth. To answer this, we transfected NT control and Sept-7 KD cells with paxillin-mCherry and imaged cells live using TIRFM (Videos S3 and S4). Examination of time-lapse movies revealed that in NT siRNA expressing cells, paxillin-mCherry localized to peripheral and perinuclear FAs and in peripheral FAs showed formation, turnover, and maturation dynamics similar to previous published reports^39^ (Video S3). Unlike peripheral FAs, perinuclear FAs in NT control MEFs were more stable with longer lifetimes and fewer new perinuclear FAs forming during the course of 10–20 min of image acquisition. In fact, perinuclear FAs formed prior to starting of acquisition lasted for longer than 10 min before disassembling. In addition, we also observed that several of these perinuclear FAs nucleated, formed and matured in the perinuclear area instead of being formed in the lamellipodia and maturing and stabilizing over time toward the middle of the cell (Video S3). In contrast, consistent with immunostaining data, in Sept-7 KD MEFs, paxillin-mCherry localized to peripheral FAs but was either completely absent in the perinuclear regions or present only in small perinuclear FA-like structures (Video S4). While we couldn’t observe any differences in peripheral FA dynamics within the temporal resolution of our acquisition, the existing perinuclear FAs in Sept-7 KD cells disassembled rapidly compared to NT controls. In addition, we observed formation of several perinuclear paxillin puncta during the course of 10–20 min of image acquisition in the perinuclear region that disassembled rapidly instead of maturing into larger perinuclear FAs. To quantify these dynamics, we used kymograph-based analysis and measured perinuclear FA formation rate and its observable average lifetime (Figure 3E). This analysis confirmed that loss of Sept-7 resulted in a slight increase in the formation rate of small perinuclear paxillin positive puncta, and a significant reduction in its minimum lifetime compared to NT controls. In contrast to perinuclear FAs, we couldn’t detect any significant alterations in the dynamics of peripheral FAs within the temporal resolution of our acquisition (Figure 3F). Taken together, our data show that ECM-binding dependent Sept-7 bundles promote the stabilization of perinuclear FAs by increasing the maturation rate and lifetime of perinuclear FAs in adherent cells.

Video S3. Live cell dynamics of MEFs transfected with non-targeting siRNA, related to figure 3 TIRFM time-lapse imaging of MEFs transfected with NT siRNA control for 48 h and paxillin-mCherry (gray) prior to imaging. Images taken every 10 s (elapsed time shown in min:s). Inset shows perinuclear FA dynamics. LUT inverted

Video S4. Live cell dynamics of MEFs transfected with Sept-7 targeting siRNA, related to figure 3 TIRFM time-lapse imaging of MEFs transfected with Sept-7 targeting siRNA for 48 h and paxillin-mCherry (gray) prior to imaging. Images taken every 10 s (elapsed time shown in min:s). Inset shows perinuclear FA dynamics. LUT inverted

### Integrin-activation dependent Sept-7 regulation enhances sensitivity of cells to changes in ECM cues and contributes to their ECM remodeling function

**Figure 4:**
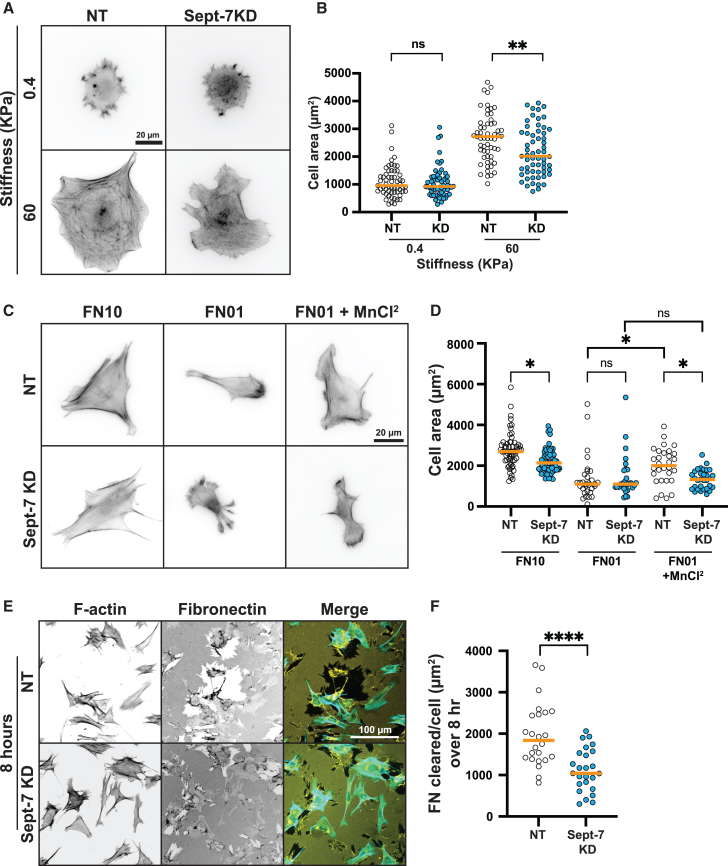
Integrin-activation dependent Sept-7 regulation enhances sensitivity of cells to changes in ECM cues and contributes to their ECM remodeling function (A) Representative wide-field fluorescence images of MEFs transfected with non-targeting or Sept-7 siRNA plated on soft (0.4 kPa) or stiff (60 kPa) polyacrylamide gels and probed for F-actin; scale bar, 20 μm. (B) Quantification of cell area of NT or Sept-7 KD MEFs on soft or stiff polyacrylamide gels, *n* = 3 replicates, 59–61 cells. (C) Wide-field fluorescence images showing NT and Sept-7 KD MEFs stained for F-actin and plated on 10 μg/mL FN, 0.1 μg/mL FN, and 0.1 μg/ml FN + MnCl^2^; scale bar, 20 μm. (D) Quantification of cell area for NT and Sept-7 KD MEFs plated on 10 μg/mL FN, 0.1 μg/mL FN, and 0.1 μg/ml FN + MnCl^2^, *n* 2–3 replicates, 29–59 cells. (E) Wide-field fluorescence images showing NT and Sept-7 KD MEFs plated for 8 h on 10 μg/mL FN and co-stained with F-actin (cyan), and FN (yellow); scale bar, 100 μm. (F) Quantification of the average area of FN clearance per cell in NT and Sept-7 KD cells, 2 replicates, *n* = 555–645 images. All statistics performed with Kruskal-Wallis and Dunn’s multiple comparisons test or Mann-Whitney test, ∗∗∗∗*p* < 0.0001, ∗∗∗*p* < 0.001, ∗∗*p* < 0.01, ∗*p* < 0.05, ns, not significant, orange horizonal lines show medians.

Our results show that physical cues from the ECM can regulate the localization and morphology of Sept-7 structures on the ventral surface of cells and that this is partially mediated through integrin activation. Additionally, we found that the FA localization-specific architecture of Sept-7 regulates the stability and dynamics of specific sub-populations of FAs. This led us to hypothesize that ventral Sept-7 structures are critical for cellular functions that depend on FA-mediated sensing of changes in the ECM which rely on changes in integrin activation level. To test this, we first investigated the role of Sept-7 in sensing changes in ECM rigidity by plating MEFs on PAA gels with stiffness of 0.4KPa and 60KPa and measuring cell area across these conditions (Figures 4A and 4B). We verified that increasing the stiffness from 0.4KPa to 60KPa did indeed result in changes in Sept-7 organization by altering the anisotropy of perinuclear Sept-7 structures on the ventral surface of NT control MEFs (Figure S5A). Technical issues related to imaging through the hydrogel precluded us from measuring any changes in peripheral Sept-7 structures. The change in perinuclear Sept-7 anisotropy however as expected correlated with increase in cell spread area on 60KPa compared to 0.4KPa PAA gels (Figure 4B). In contrast, we found that while Sept-7 KD MEFs spread to the same size as NT control cells on 0.4KPa gels, the cell spread area was significantly smaller on 60KPa (Figure 4B). This was consistent with a previous study showing reduction in sensitivity of Sept-7 depleted cancer-associated fibroblasts to changes in ECM stiffness.^33^ This suggests a role for ECM stiffness-dependent changes in Sept-7 organization in promoting sensitivity of cellular response to changes in ECM stiffness.

Since our results show that elongation and FA-localization of peripheral Sept-7 structures is sensitive to changes in ECM density and levels of integrin activation (Figure 2), we next tested if this pool of Sept-7 was critical in allowing cells to respond to changes in ECM density (haptosensing).^45^^,^^46^ We plated NT control and Sept-7 KD MEFs on dishes either coated with 0.1 μg/mL FN (low Sept-7-peripheral paxillin co-localization; punctate Sept-7 peripheral structure) or on 10 μg/mL FN (high Sept-7-peripheral paxillin co-localization; elongated peripheral Sept-7 structure) and stained the cells with phalloidin for measuring cell area 4 h after plating (Figure 4C). Imaging for cell-spread area showed that both NT control and Sept-7 KD MEFs failed to spread properly on 0.1 μg/mL FN while increasing the FN density to 10 μg/mL FN led to increased spreading and formation of stress fibers across the cell in the NT controls and increased spreading without clear stress fibers in the Sept-7 KD cells (Figure 4C). Quantification of cell area confirmed that similar to changes in stiffness, Sept-7 KD MEFs were similar to NT controls on 0.1 μg/mL FN, but significantly smaller on 10 μg/mL FN, resulting in an overall reduction in sensitivity to changes in ECM density in Sept-7 KD cells (Figure 4D). To further test if this was due to integrin-activation specific regulation of peripheral Sept-7 structures, we treated both cell types plated on 0.1 μg/mL FN with 1 mM MnCl_2_ which promotes elongation of Sept-7 in peripheral FAs (and has no effect on perinuclear structures) and found that while it led to an increase in cell spread area in NT control MEFs, it had no effect on Sept-7 KD MEFs (Figure 4D). Taken together, these results show that Sept-7’s association with perinuclear and peripheral FAs is important in sensing changes in ECM density. Our results also suggest a role for peripheral Sept-7 structures in increasing sensitivity of cellular responses to changes in integrin activation level.

To investigate the role of perinuclear Sept-7 structures and due to the known perinuclear localization of fibrillar FAs,^47^^,^^48^ we asked if Sept-7 plays a role in ECM remodeling. To test this, we first plated NT control and Sept-7 KD MEFs on 10 μg/mL FN and then fixed and immunostained the cells for FN and F-actin 8 h after plating (to allow for ECM clearing in 2D). We then used wide-field florescence microscopy to quantify the area of cleared FN at 8 h (Figure 4E). We observed areas of FN clearance in both conditions but quantification of the average area of FN cleared per cell after 8 h revealed a significant drop in ECM clearance in Sept-7 KD cells (Figure 4F). To test if this loss of FN remodeling was due to impeded cell migration or the ability of cells to remodel bound ECM, we tracked the migration of NT and Sept-7 KD cells labeled with SiR-DNA over 12 h. Quantification of cell migration speed revealed a slight reduction in migration speed in Sept-7 KD cells compared to NT control cells and no change in persistence or forward progress due to loss of Sept-7 (Figure S5B). However, the differences in cell migration speed was insufficient to account for the differences in cleared FN between control and Sept-7 KD cells. This suggests a critical role for Sept-7 in ECM remodeling through either regulation of perinuclear FAs or through supporting ECM-dependent optimal cell spreading and cell migration.

Taken together, these data show critical roles for the distinct ventral pools of Sept-7 in ECM and integrin-dependent cellular processes with ECM-dependent integrin activation mediated changes in peripheral Sept-7 resulting in increased sensitivity in cellular responses to ECM density and integrin activation, while perinuclear Sept-7 structures being more critical for cellular responses to changes in ECM stiffness while also promoting ECM remodeling.

## Discussion

Our results here show that Sept-7 is an FA-associated protein that gets assembled into different structures in and in proximity of FAs in an ECM and integrin activation dependent manner in fibroblasts. In addition to differences in their architecture, these ventral Sept-7 structures also differentially localize to FA subpopulations with bundles of Sept-7 localizing to the back of perinuclear FAs while elongated FA-like Sept-7 structures localize to the core of peripheral FAs. Besides localizing to FA subpopulations, our results show an important role for Sept-7 in regulating these subpopulations. In peripheral FAs, Sept-7 seems to promote FA elongation, while in perinuclear FAs, Sept-7 not only increases maturation rate but also contributes to the stabilization and growth of these FAs. Functionally, we find that downregulation of Sept-7 expression results in fibroblasts losing their sensitivity to distinct changes in ECM cues, with peripheral Sept-7 structures playing a critical role in sensing of changes in ECM density while perinuclear Sept-7 being more important for ECM stiffness sensing. In addition, Sept-7 also plays an important role in promoting fibroblast cells ability to remodel the ECM potentially through perinuclear Sept-7. Collectively, these results show that Sept-7 is an important protein of the FA adhesome that via assembly of different structures determines the sensitivity of fibroblasts to sense changes to their ECM environment and regulate their functional response to changes in ECM cues.

Previous studies on the role of septins in regulating FAs and mechanotransduction have attributed many of these functions to its ability to interact and regulate F-actin in the vicinity of FAs.^32^^,^^33^^,^^34^ Through this potential mechanism, septins have been shown to regulate several properties of the FA including stabilization and elongation of peripheral FAs.^32^ A more recent study showed that septins can also target non-centrosomal microtubules to FA sites to drive FA disassembly.^28^ However, the precise mechanisms of this regulation are still not clear, since septins were thought to be excluded from FAs.^31^^,^^32^ Here, our data adds more detail to the mechanisms by showing that Sept-7 is in the core of peripheral FAs where it co-localizes with paxillin and F-actin. While further investigations on the mechanisms of FA recruitment of Sept-7 need to be done, a recent study using pull-down and mass spectrometry identified talin as one possible binding partner of Sept-7.^35^ Talin is a large multi-domain FA protein that links the cytoplasmic tails of integrins to F-actin. Under mechanical forces, talin opens up to reveal a large number of binding sites for other FA proteins.^49^ Investigating whether Sept-7 is one such FA protein will be subject of further studies. Other potential binding partners for septins include vinculin, LMO7 and ZNF185 which were identified in a separate proteomics study investigating the Sept-9 interactome in human fibroblasts.^50^ A more recent study also found Sept-7 as a potential interactor with Hic-5 and paxillin in U2OS cells which is consistent with our localization data.^51^

Our results here also lead to new questions about assembly of ventral septin structures. Septins form filaments by annealing hetero-oligomers which further form higher-order structures such as bundles and rings either through end-to-end binding or through lateral stacking.^52^ Interestingly, Sept-7 is a component that is present in both fundamental units of septin oligomers, hexamers, and octamers and thus are an integral part of all higher order septin structures in a cell.^53^^,^^54^ Our data on the ventral cell surface shows Sept-7 in two different forms, elongated FA-like in the peripheral FAs and longer bundles in the perinuclear region associated with perinuclear FAs. We show here that these structures while being dependent on the ECM have different reliance and sensitivity to integrin activation and ECM binding. The different form and mechanism of formation suggests distinct ECM-dependent mechanisms of regulation of septin architecture on the ventral cell surface. Previous studies have shown that perinuclear septin bundles interact with perinuclear ventral actin stress fibers via Cdc42EP3 that stabilizes F-actin.^33^^,^^55^ Through ECM-dependent direct regulation of ventral stress fibers, it is possible that these perinuclear septin structures get formed and organized. Since we find that septin bundles are spatially confined to the perinuclear ventral surface and excluded from peripheral regions, this suggests additional regulation of this mechanism such as through formins that localize to the perinuclear region or through other ventral stress-fibre binding proteins. Our results also show that Sept-7 bundles specifically target ventral actin stress fibers and seems to have no effect on the actin cap on top of the nucleus even though Sept-7 can localize there suggesting that the mechanisms of Sept-7 bundles are potentially restricted to the ventral surface only. It is also possible that perinuclear Sept-7 bundles are organized either directly by perinuclear FAs or through a continuous process of FA maturation from the peripheral FAs to perinuclear FAs. However, we find that on 2D surfaces, several of the perinuclear FAs form *de novo* in the perinuclear region instead of from growing and maturing peripheral FAs. The timescales of our movies also does not currently allow us to see if Sept-7 bundles form from retrograde motion and annealing of peripheral Sept-7 structures, though this is an interesting possibility that is easily testable with better probes and imaging systems. Unraveling any specific perinuclear FA-dependent mechanism would also require detailed identification of what the perinuclear FAs are. We find that the perinuclear FAs in our study are not restricted to fibrillar adhesions and are enriched for most canonical FA proteins. Thus, detailed compositional analysis of perinuclear FAs associated with Sept-7 bundles along with time-lapse imaging of Sept-7 dynamics from the periphery to the perinuclear regions in the future along with studies on actin regulatory proteins downstream of ECM binding should provide more detailed information about the regulation of perinuclear Sept-7.

Even less is known about the regulation of FA-like elongated peripheral Sept-7 structures. Their dependence on ECM-binding dependent integrin activation but not on integrin-mediated signaling suggests that these structures are directly or indirectly dependent on binding to FA proteins that undergo conformational changes at high forces or are associated with highly mature FAs. With recent evidence of peripheral septin structures allowing targeting of microtubules to peripheral FAs, as well as earlier studies on peripheral septins affecting FA dynamics, coupled with our data on septins in the core of the peripheral FAs, these studies show an important role for septins in the leading edge of cells.^28^^,^^32^ Whether our core-peripheral FA Sept-7 structures are distinct from proximal to FA structures from the other studies needs to be investigated more. We also observed several Sept-7 structures that do not colocalize with peripheral FAs, again suggesting more distinct sub-pools of septins structures at the edge of the cell. Here, we also would like to highlight that we find peripheral FAs that are not positive for elongated Sept-7 structures, which from the FA perspective suggests again heterogeneous populations of FAs, even within the periphery of the cell. What the function of these FAs specifically are, how sept-7 or other septins affect these sub-pools and how septins gets recruited to FA sites will be the subject of future studies.

This study shows that loss of Sept-7 and the resultant loss in perinuclear FA function results in diminished ability of fibroblasts to sense and respond to biophysical changes in the ECM. Here, we find that integrin-activation dependent peripheral Sept-7 structures contributes to this sensitivity. A number of different mechanisms have been suggested by which cells tune their sensitivity and specificity to changes in ECM cues.^56^ Our data here along with previous studies show that part of this mechanism relies on FA heterogeneity and that septins can contribute to making these FAs functionally and compositionally distinct.^57^^,^^58^^,^^59^ However, very little is known about the specific roles of FA subpopulations in addition to their compositional and organizational differences. Our data show that Sept-7 plays a critical role in regulating perinuclear FAs and has a subtler but significant effect on peripheral FA morphology though previous studies have shown a much more important role for septins in regulating peripheral FAs.^32^ These differences are likely due to the differences in contractility and function of fibroblasts compared to MDCK cells in some of the other studies and a more detailed analysis with high spatiotemporal resolution needs to be done in fibroblasts to look at the precise role of Sept-7 in peripheral FAs. For perinuclear FAs, since the Sept-7 structures that coincides with this subpopulation are the perinuclear bundles, this suggests an overall organizational difference between perinuclear and peripheral FAs. As mentioned above, it is also likely that perinuclear FAs in our system are not one population but a few different populations of FAs with more specific functions that are currently unknown. Due to the effect of these perinuclear FAs on ECM remodeling, it is tempting to speculate that these perinuclear FAs are fibrillar adhesions that originate at the medial margins of classic FAs, changing protein composition and moving to the center of the cell.^60^ Paxillin however is thought to excluded from fibrillar adhesions, which instead contain tensin-1 that links F-actin to the cytoplasmic tail of integrins.^48^^,^^61^ This shows that while the perinuclear FAs in our study are not fibrillar adhesions, there are some functional overlaps that needs to be investigated further.

Lastly, similar to ECM-density dependent regulation of FA dynamics and its coupling to the actin cytoskeleton through the Arp2/3 complex, our data shows an ECM density-dependent regulation of Sept-7 morphology and localization at FAs. Furthermore, the sensitivity of peripheral Sept-7 structures to ECM density and integrin activation at peripheral FA sites suggests that Sept-7 may have a critical role at the leading edge, perhaps even at nascent adhesions, which are considered along with the lamellipodia as haptosensing organelle.^39^^,^^45^ This coupled to the previous studies on Sept-9 In FA maturation at the leading edge not only reaffirms the important role of septins in cell motility but also in ECM-dependent regulation of other cellular processes.^32^

In conclusion, our work proposes spatially and mechanistically distinct roles in ECM-mediated cellular processes for ventral Sept-7 in the dynamics and function of perinuclear and peripheral FA subpopulations. More details about these distinct roles of ventral Sept-7 requires further studies including understanding mechanisms of their recruitment to the FAs and their detailed structural organization within these distinct pools.

### Limitations of the study

Our study here identifies two spatially distinct Sept-7 structures localized to FAs at either the perinuclear or peripheral region of the cell. While these structurally and dynamically distinct Sept-7 localizations suggest that the role of Sept-7 at these separate FA sites differ, our study currently does not provide clear evidence of this. While we show differences in sensitivity of the 2 pools to ECM density and integrin activation levels, more mechanistic details and discovery of molecular mechanisms which would allow for selective disruptions of these 2 pools would allow better insight into these distinct roles.

Additionally, while this study shows that both Sept-7 and Sept-9 localize at both FA subsets, suggesting the presence of septin oligomers, details of structural organization at these ventral regions and if there are indeed differences is not shown in this study. This is particularly critical in understanding the role of septins where components building the oligomer can affect the overall organization of septins on the ventral surface.^27^ The structural details of the distinct pools as well as their mechanisms of formation and regulation will be explored in future studies.

## Resource availability

### Lead contact

Further information and request for reagents and resources should be addressed to, and will be fulfilled by, the lead contact, V.S. (vinay.swaminathan@med.lu.se).

### Materials availability

This study did not generate new unique reagents.

### Data and code availability

• Data: The raw excel data sheets for all quantification have been deposited on Mendeley Data and are publicly available as of the date of publication. Accession numbers are listed in the key resources section.
• Code: The code used for localization analysis has been deposited on Mendeley Data and will be publicly available as of the date of publication. Accession numbers are listed in the key resources section.
• All other items: any additional information needed for data presented in this paper is available from the lead contact upon request.

## Acknowledgments

We thank Dr. Johan Malmström, Dr. Sebastian Wasserstrom and all the members of laboratory of cell and molecular mechanobiology (LCMM) for their discussion and support. The Sept-7 YFP plasmid was a kind gift from Dr. Helge Ewers at the Freie Universität Berlin. Johannes Kumra Ahnlide and Valeriia Grudtsyna are specially acknowledged for the help in developing code and maintaining image storage servers. Lund University Bioimaging Center (LBIC) at Lund University is gratefully acknowledged for providing experimental resources. This research was funded by the 10.13039/501100004063Knut and Alice Wallenberg Foundation (W.S. and V.S.), Wallenberg Centre for Molecular Medicine, Lund); 10.13039/501100002794Cancerfonden (VS, 19 0445 Pj and 22 2398 Pj Projekt grant) and The Royal Physiographic Society of Lund (W.S., App: 43178).

## Author contributions

Conceptualization, W.S. and V.S.; Methodology, W.S. and V.S.; Software, P.N.; formal analysis, W.S., R.G., and P.N.; Investigation, W.S., S.P., and R.G.; Writing – Original Draft, W.S. and V.S.; Writing - Review and Editing, W.S. and V.S.; Visualization, W.S. and V.S. Funding acquisition, V.S.; Supervision, V.S.

## Declaration of interests

The authors declare no competing interests.

## STAR★Methods

### Key resources table

**Table:** 

REAGENT or RESOURCE	SOURCE	IDENTIFIER
**Antibodies**		

Septin7 rabbit polyclonal primary antibody	Thermofisher scientific	Cat#PA5-54755; RRID: AB_2647128
GAPDH rabbit polyclonal primary antibody	Sigma-Aldrich	Cat#PLA0125; accession no: NP_002037.2
Starbright™ Blue 520 Goat Anti-Rabbit IgG secondary antibody	Biorad	Cat#12005869
Starbright™ Blue 700 Goat Anti-mouse IgG secondary antibody	Biorad	Cat#12004158
Mouse anti-paxillin primary antibody	BD Bioscience	Cat#610052; RRID:AB_397464
Raibbit anti-fibronectin primary antibody	Sigma-Aldrich	Cat#F3648; Uniprot accession:P02751
Mouse anti-vinculin primary antibody	Sigma-Aldrich	Cat#V4505; Uniprot accession:P18206
Mouse anti-FAK primary antibody	Sigma-Aldrich	Cat#06–543; Uniprot accession:Q05397
Rat anti-mouse CD29 (9EG7) primary antibody	BD Biosciences	Cat#553715; RRID: AB_395001
Goat anti-mouse IgG 647 secondary antibody	Invitrogen	Cat#A21235; RRID: AB_2536183
Goat anti-rabbit IgG 568 secondary antibody	Invitrogen	Cat#A11010; RRID: AB_143157
Alexa Fluor 488 phalloidin	Invitrogen	Cat#A12380
Rabbit anti-tensin1 primary antibody	Sigma-Aldrich	Cat#SAB4200283; RRID: AB_10897435

**Chemicals, peptides, and recombinant proteins**		

DMEM	Gibco	Cat#61965026
Fetal bovine serum	Gibco	Cat#10270106
Penicillin/streptomycin	Gibco	Cat#15140122
Bovine serum albumin	Sigma-Aldrich	Cat#A7906
RIPA lysis buffer	Thermo-fisher Scientific	89901
Fibronectin	Sigma Aldrich	Cat#0895; Uniprot accession: P02751
Poly-L-lysine	Sigma Aldrich	Cat#P4707
Formaldehyde	Thermo Fisher Scientific	Cat#28906; CAS:50-00-0
Triton X-	alfa-Aesar	Cat#A16046; CAS:9002-93-1
Glycine	Sigma Aldrich	Cat#50046; CAS:56-40-6
Mounting media	Thermo Fisher Scientific	Cat#P36980
*N*-hydroxysuccinimide (NHS)	Thermo Fisher Scientific	Cat#24500; CAS:56-40-6
40% Acrylamide Solution	Bio-Rad	Cat#1610140; CAS:79-06-1
2% Bis-acrylamide solution	Bio-rad	Cat#1610142
TEMED	Bio-rad	Cat#1610800; CAS:110-18-9
Amonium persulphate (APS)	Sigma-Aldrich	Cat#09913; CAS:7725-54-0
Laemmlli Sample Buffer	Bio-rad	Cat#1610747
Pierce Protease and Phosphatase Inhibitor	Thermo Fisher Scientific	Cat#A32961
Manganese cloride	Sigma-Aldrich	Cat#M1787; CAS:773-01-5
PlusOne Repel-Silane ES	Sigma-Aldrich	Cat#GE17-1332-01; CAS:556-67-2
Lithium phenyl-2,4,6-trimethylbenzoylphosphinate (LAP)	Sigma-Aldrich	Cat#900889; CAS:85073-19-4
HEPES	Thermo Fisher Scientific	Cat#15630106
Fetal bovine serum	Gibco	Cat#10270106
Penicillin/streptomycin	Gibco	Cat#15140122
Laemmli sample buffer	Bio-Rad	Cat#1610747
Bovine serum albumin	Sigma-Aldrich	Cat#A7906
RIPA lysis buffer	Thermo-fisher Scientific	89901
Fibronectin	Sigma Aldrich	Cat#0895; Uniprot accession: P02751
Poly-L-lysine	Sigma Aldrich	Cat#P4707
Formaldehyde	Thermo Fisher Scientific	Cat#28906; CAS:50-00-0
Triton X-	alfa-Aesar	Cat#A16046; CAS:9002-93-1
Glycine	Sigma Aldrich	Cat#50046; CAS:56-40-6
3-aminopropyltrimethoxysilane	Merck	Cat#281778; CAS:13822-56-5
Blebbistatin	Merck	Cat#203390; CAS:674289-55-5
FAK inhibitor	Merck	Cat#PF-573228; CAS:869288-64-2

**Critical commercial assays**		

Lipofectamine™ 3000 Transfection Reagent	Thermo Fisher Scientific	Cat#L3000001

**Deposited data**		

Deposited excel files for all quantification	Mendelay data	https://doi.org/10.17632/wvnywnb7rn.1
Deposited Julia code for focal adhesion colocalization analysis	Mendelay data	https://doi.org/10.17632/wvnywnb7rn.1

**Experimental models: Cell lines**		

Mouse embryonic fibroblasts (MEFs)	Gift from Michael Davidson lab, Florida state university	

**Oligonucleotides**		

ON-TARGETplus mouse Sept7 (235072) siRNA smartpool	Dharmacom	Cat# I-042160-01-0005; Accessions: NM 001205367, NM009859, XM 006510190, XM 006510191, XM 006510192, XM 006510193
ON-TARGETplus Non-targeting Control Pool	Dharmacom	Cat# D-001810-10-05

**Recombinant DNA**		

Paxillin-mCherry plasmid	Gift from Michael Davidson lab, Florida state university	
Sept-7-YFP plasmid	Gift from the lab of Helge Ewers, Germany	

**Software and algorithms**		

ImageJ	N/A	https://imagej.nih.gov/ij
GraphPad Prism 10.2	GraphPad Software, CA, USA	https://www.graphpad.com
R programming language (1.2.5033)	N/A	https://www.R-project.org/
Julia (1.10.2)	N/A	https://julialang.org/

### Experimental model and study participant details

This study used mouse embryonic fibroblast cells which to our knowledge have not been authenticated. The cells were not tested for mycoplasma contamination. The culture conditions for these cells are listed in the method details section.

### Method details

#### Cell culture

Mouse embryonic fibroblasts (MEFs) were a generous gift from Dr Clare Waterman. Cells were cultured in DMEM (Gibco, 15140122) supplemented with 10% FBS (Gibco, 10270106), and penicillin/Streptomycin (Gibco, 15140122) at 37^°^C and 5% CO_2_.

#### Western blotting

Cells were placed on ice and rinsed with PBS before being lysed for 10 min in Ripa buffer (Thermo fisher Scientific, 89901) supplemented with Protease and phosphatase inhibitors (Thermo fisher Scientific, A32961) whereby cells were scraped and lysates collected. The lysate was spun at 12000 rpm for 10 min and the supernatant was collected. Denaturing of the proteins was performed using 4% Laemmilli loading buffer (Bio-rad,1610747) at 95^°^C for 10 min followed by protein separation using gel electrophoresis. Proteins were transferred onto PVDF membranes using the Bio-rad *Trans*-blot kit (Bio-rad, 1704274). Next, membranes were blocked using 3% BSA in TBS-T for 1 h at room temperature. Membranes were incubated overnight with primary antibodies Sept-7 (Thermo Fisher Scientific, PA5-54755, 1:400 concentration), and GAPDH (Sigma-Aldrich, PLA0125, 1:3000 concentration) at 4^°^C with continuous gentle rocking. Membranes were then washed 3 times in TBS-T before being probed with Starbright Blue 520 Goat Anti-Rabbit IgG (Biorad, 12005869) or Starbright Blue 700 Goat Anti-Mouse IgG (Biorad, 12004158) at 1:2500 concentration in 3% BSA in TBS-T at room temperature for 1 h. After washing 3 times in TBS-T the membranes were imaged using a chemiDoc MP system (Bio-rad).

#### Immunostaining

Cells were fixed in 4% paraformaldehyde (Thermo scientific, 28906) in cytoskeleton buffer (CB) for 20 min at 37^°^C . To permeabilize the cell membrane 0.5% Triton X-(Alfa Aesar, A16046) in CB was added for 5 min. Cells were then washed at room temperature (RT) with 0.1 M Glycine (Sigma, 50046-250G) in CB for 10 min and 3 times 5 min in TBS with gentle rocking, and then blocked with 2% BSA in TBS-T for 1 h. Incubation of Primary Abs: Paxillin 1/400 (BD Bioscience, 610052), Sept7 1/400 (Thermo scientific, PA5-54755), FN 1/400 (Sigma-Aldrich, F3648-100UL), vinculin 1/400 (Sigma-Aldrich, V4505), FAK 1/400 (Sigma-Aldrich, 06–543), 9EG7 1/400 (BD Biosciences, 553715), tensin-1 1/400 (Sigma-Aldrich, SAB4200283) in 2% BSA in TBS-T was performed overnight at 4^°^C . Cells were washed in TBS-T 3 × 5 min with gentle rocking and then incubated with secondary Ab: goat anti-mouse IgG 647 nm 1/400 (invitrogen, A1101), goat anti-rabbit IgG 568 nm 1/400 (Invitrogen, A11010) and phalodin 488 1/400 (Invitrogen, A12380) in 2% BSA in TBS-T for 1 h in the dark at RT and then washed 3 × 5 min in TBS-T and finally either mounted on slides or left in TBS for imaging.

#### Integrin activation assay

Glass bottom dishes were coated with 0.1 or 10 μg/mL FN in PBS, or Poly-L-lysine (PLL) (Sigma-Aldrich, P4707), for 1 h at 37^°^C . Dishes were then rinsed with PBS and left overnight in 2% BSA in TBS-T and then rinsed with PBS. Cells were then plated on the FN or PLL coated dishes for 4 h at 37°C in DMEM, 1 mM MnCl^2^ (Sigma-Aldrich, M1787) was added to appropriate dishes 2 h after plating. Cells were then fixed for immunostaining (see above method).

#### Inhibitor assay

Glass bottom dishes were coated with 10 μg/mL FN in PBS for 1 h at 37^°^C . Dishes were then rinsed with PBS and left overnight in 2% BSA in TBS-T and then rinsed with PBS. Cells were then plated for 4 h at 37°C in DMEM, 50 μm blebbistatin (Merck, 203390) or 1μM FAK inhibitor (Merck, PF573228) was added to appropriate dishes 2 h after plating. Cells were then fixed for immunostaining (see above method).

#### ECM stiffness assay

##### Functionalization of coverslips

Coverslips were submerged in 0.2M hydrogen chloride (HCl) and left rocking at room temperature overnight. After washing 5 × 30 min with H_2_O, they were washed with sodium hydroxide (NaOH) at RT for 1 h at 37^°^C . After washing 5 × 30 s in H_2_O the coverslips were washed for 1 h at RT in 1:100 3-aminopropyltrimethoxysilane in MQ water with gentle rocking, followed by 1 h washing with 1:140 of cold 70% glutaraldehyde in PBS with gentle rocking. Finally wash 3 × 10 min in MQ water with gentle rocking, place on kim wipes and leave to dry overnight at RT.

##### Preparing gels

Gels of different elastic moduli were prepared using reagent volumes from the table below. Combine MQ water, 10x PBS, and 2% Bis-acrylamide (Bio-rad, 1610142) into an Eppendorf tube. Next add 1% TEMED (Bio-rad, 1610800) and 40% acrylamide (Bio-rad, 1610140) to the tube containing the bis-acrylamide solution. Next, a degas chamber to degas the bis-acrylamide solution, and APS (Sigma-Aldrich, 09913) in a separate tube for 30 min, the solutions should be kept in the dark. While the reagents are degassing a large glass dish was cleaned with detergent and water followed by ∼800 μL PlusOne Repel-Silane ES (Sigma-Aldrich, GE17-1332-01) and left to dry. Working in a fume hood and in the dark APS was added to the bis-acrylamide solution and mixed well, but careful not to introduce air bubbles into the mixture. 25μL drops of the gel solution were pipetted onto the rainX-treated glass dish and functionalized coverslips were placed onto the drops and left to dry for 1 h. The functionalized coverslips were washed in MQ water for 10 min, then the MQ water was replaced and stored at 4^°^C ready for functionalization.

**Table:** 

Elastic modulus (Pa)	Water (mL)	40% acrylamide (mL)	2% Bis-acrylamide(ml)	10x PBS (mL)	1% Temed (mL)	1% APS (mL)
400	0.7	0.075	0.025	0.1	0.05	0.05
60,000	0.3	0.25	0.25	0.1	0.05	0.05

##### Gel functionalization

To functionalize gels, 480 μL H_2_O, 100 μL HEPES (Thermo Fisher Scientific, 15630106) and 50 μL 0.2% bis-acrylamide was mixed in an Eppendorf tube and wrapped in foil and degassed for 30 min 10 mg of LAP (Sigma-Aldrich, 900889) was dissolved in 1 mL H_2_O and 3 mg of NHS (Thermo Fisher Scientific, 24500) in 1 mL of 50% ethanol and kept covered with foil and on ice. A 12 well plate with 1 mL H_2_O in each well and a tube of 0.9% NaCl was placed on ice. Large coverslips were washed with soap and water, wiped dry with Kimwipes rain-X was added for 5 min, after which they were rinsed with ethanol and H_2_O and then dried out completely. Next, working in a fume hood and in the dark 30 μL LAP and 340 μL NHS was added to the degassed solution and mixed carefully to avoid air bubbles. 40 μL drops of functionalization solution was pipetted onto rain-x coated coverslips. Premade coverslips with gels were placed onto the functionalization drops and placed under UV light for 15 min. Functionalized gels were then moved to the 12 well plates containing H_2_O shaking on a medium speed for 5 min. Gels were then washed 2 × 5 min with ice-cold 0.9% NaCl shaking on medium speed. Gels can be stored ready for use in 0.9% NaCl at 4^°^C .

##### Transfection

For plasmid transfection cells were seeded in 6-well plates. Plasmids were incubated in serum free media (SFM) with Lipofectamine 3000 transfection reagent (Thermo Scientific, L3000001) at either 2,5 or 5 μg/mL concentrations (single or co-transfection respectively) for 20 min before adding to cells. Plasmid used were paxillin-mCherry and/or Sept-7-YFP. For siRNA transfection 20p.m. non-targeting (Dharmacon, D-001810-10-05), or Sept-7 targeting (Dharmacon, L-042160-01-005) siRNA was incubated in SFM with Lipofectamine 3000 transfection reagent for 20 min before adding to cells. In both plasmid and siRNA cases cells were used 48 h after transfection.

##### Total internal reflection microscopy (TIRFM)

Images were acquired using total internal reflection microscope on a Nikon Eclipse Ti microscope with TIRF APO 100 × 1.49 N.A. objective. Laser lines used were 488, 561, and 647 nm and emission and excitation filters were: GFP (mirror: 498–537 nm and 565–624 nm; excitation: 450–490 nm and 545–555 nm; emission: peak 525 nm, range 30 nm) and mCherry (mirror: 430–470, 501–539, and 567–627 nm; excitation: 395–415, 475–495, and 540–560 nm; emission: peak 605 nm, range 15 nm), or Continuous STORM (mirror: 420–481, 497–553, 575–628, and 667–792 nm; excitation: 387–417, 483–494, 557–570, and 636–661 nm; emission: 422–478, 502–549, 581–625, and 674–786 nm). Images were acquired using a Teledyne Photometrics 95B 22 mm camera. For live cell imaging cells were kept at 37^°^C and images were captured every 10–30 s over a 20 min time frame.

##### Structured illumination microscopy (SIM)

Image acquisition was performed using a Nikon N-SIM microscope with an LU-NV laser, and a CFR SR HP apochromat TIRF 100× oil objective (N.A: 1.49), 488 and 568 laser lines were used for F-actin Sept-7 respectively. An ORCA-flash 4.0 sCMOS camera (Hamamatsu Photonics K.K) was used and the images were reconstructed using in-built Nikon SIM software on NIS elements AR (NIS-A 6D and N-SIM analysis).

##### Wide-field fluorescence imaging

Image acquisition was performed using a Nikon Eclipse Ti2 microscope with an APO 20 × 0.75 N.A. objective. Excitation and emission light was passed through a FITC (Exc. 457-487nm, Em. 503-538nm) or Cy5 (Exc. 590-645nm, Em. 659–736 nm) Semrock filter cube. Images were acquired on a Nikon DS-Qi2 CMOS camera. For live cell imaging, an environmental chamber (Okolab) was used to keep samples in a humidified 37°C and 5% CO_2_ atmosphere. Cells were imaged every 5 min for 12 h.

### Quantification and statistical analysis

All performed in ImageJ unless stated otherwise.

#### Cell area

All cell area analysis was performed on 20x wide-field fluorescence images using manually created pipeline written in Julia (version 1.6), where Otsu thresholding was used to attain cell size.

#### Colocalization plots

Were acquired by creating line plots of the average intensity values along cross sections of perinuclear or peripheral FAs of background subtracted images. Data was normalized using $\frac{i-\min\;i}{\max[i-\min i]}$ where i denotes intensity. Curve plots were created in R using the ggplot2 package and the fit of the data was created using locally estimated scatterplot smoothing (LOESS).

#### FA and Sept-7 structure morphology

ROIs of perinuclear and peripheral FAs, and peripheral Sept-7 structures from background subtracted 100x TIRFM images were created. A median filter and Otsu thresholding was applied, and a mask created. FA sizes and numbers were then calculated using the built-in ImageJ function to analyze particles. Due to the restrictions of clean segmentation of single structures, both FA and Sept-7 structures were filtered to encompass a range of 0.20 μm^2^ < FA < 6 μm^2^. All FA data was binned so each point represents the average value of 10 random FAs.

#### FA dynamics

Kymographs were created of perinuclear FA sites from background subtracted 100x live cell movies, which were then used to measure minimum FA lifetimes and formation rates.

#### ECM remodeling

A manual threshold was used on fibronectin labeled wide-field fluorescence 20x images to segment areas of the coverslips that were clear of fibronectin signal. A mask was created, and the segmented area calculated using the analyze particle’s function, which was then divided by the number of cells in the image to give an average area of FN cleared per cell.

#### Cell migration

Cell migration analysis was performed using the TrackMate plug-in^62^ using 20x wide-field fluorescence timelapse images of cells labeled with the nuclear marker SiR-DNA (Tebubio, SC007). Cells were imaged over 12 h and cells that were continually tracked for a minimum 8hrs were included in the analysis.

#### Pearsons colocalization analysis

Cells were manually divided into an inner and outer region and saved as masks in ImageJ Fiji. Image analyses were written in Julia (1.10.2), with code available upon request. Focal adhesions were segmented based on the paxillin channel, and pixel- and morphology-based characteristics were extracted for each focal adhesion. The paxillin mask was used to extract corresponding data in other analyzed channels, such as septin and F-actin. A pixel-by-pixel Pearson correlation was calculated for each focal adhesion across paired channels. The data for each analyzed adhesion (area, intensities, and Pearson correlation) in inner and outer regions were exported and plotted in GraphPad Prism. All localization data was binned so each point represents the average value of 10 random FAs.

#### Statistical analysis

All data was analyzed using GraphPad Prism version 10 (GraphPad Software, Boston, Massachusetts USA). The specific number of replicates plus cells or FAs for each experiment is indicated as n in their respective figure legends. For all plots displaying FA area the data was binned so that each point represents 10 random FAs. Non-normally distributed data was analyzed using a Mann-Whitney U test. Kruskal-Wallis test with Dunns post hoc was used for data with multiple comparisons. Dot plots or violin plots were used for data display, with orange horizontal lines showing medians. Statistical tests used for each experiment are displayed in the corresponding figure legends with asterisks used in the plots to show statistical significance. The *p*-value ranges used for statistical significance are as follows; ∗∗∗∗*p* < 0.0001, ∗∗∗*p* < 0.001, ∗∗*p* < 0.01, ∗*p* < 0.05, ns, not significant.
