# Supplementary material for: ECM-dependent regulation of septin 7 in focal adhesions promotes mechanosensing and functional response in fibroblasts
Source: iScience. 2024 Nov 9;27(12):111355. doi: 10.1016/j.isci.2024.111355 (PMC11625310; doi:10.1016/j.isci.2024.111355)
Supplement: Document S1. Figures S1–S5 [file mmc1.pdf]

iScience, Volume *27*

Supplemental information

ECM-dependent regulation of septin 7 in focal adhesions promotes mechanosensing and functional response in fibroblasts

Wesley Sturgess, Swathi Packirisamy, Rodina Geneidy, Pontus Nordenfelt, and Vinay Swaminathan

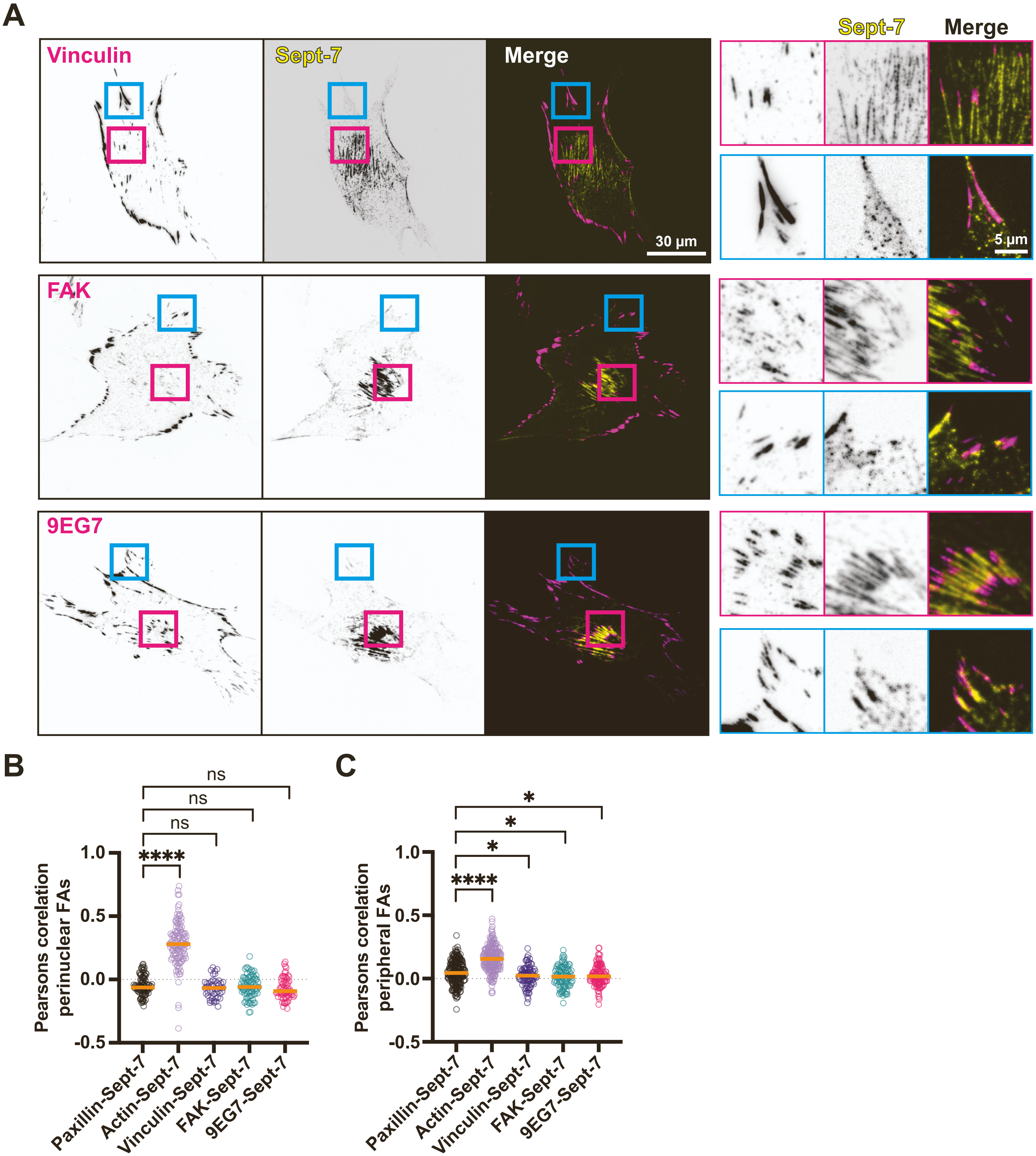

Figure S1. **Colocalization of Sept-7 with adhesion markers FAK, vinculin, and active a5b1, related to figure 1.** (A) Representative TIRFM images of MEFs stained for the FA markers (Vinculin, FAK, and active a5b1 integrin (9EG7)) (magenta), and Sept-7 (yellow) of cells plated on 10 µg/ml FN; scale bar, 30 µm. Magenta and cyan boxes highlight perinuclear and peripheral FAs respectively with insets shown on the right; scale bar, 5 µm. (B and C) Quantification of colocalization of FA markers or F-actin with Sept-7 for perinuclear (left plot) and peripheral (right plot) FAs using Pearsons's correlation, n = 1 – 3 replicates, 470 – 2270 FAs. All statistics performed using a Mann-Whitney test, **** P < 0.0001, *** P < 0.001, ** P < 0.01, * P < 0.05, ns, not significant, orange horizonal lines show medians.

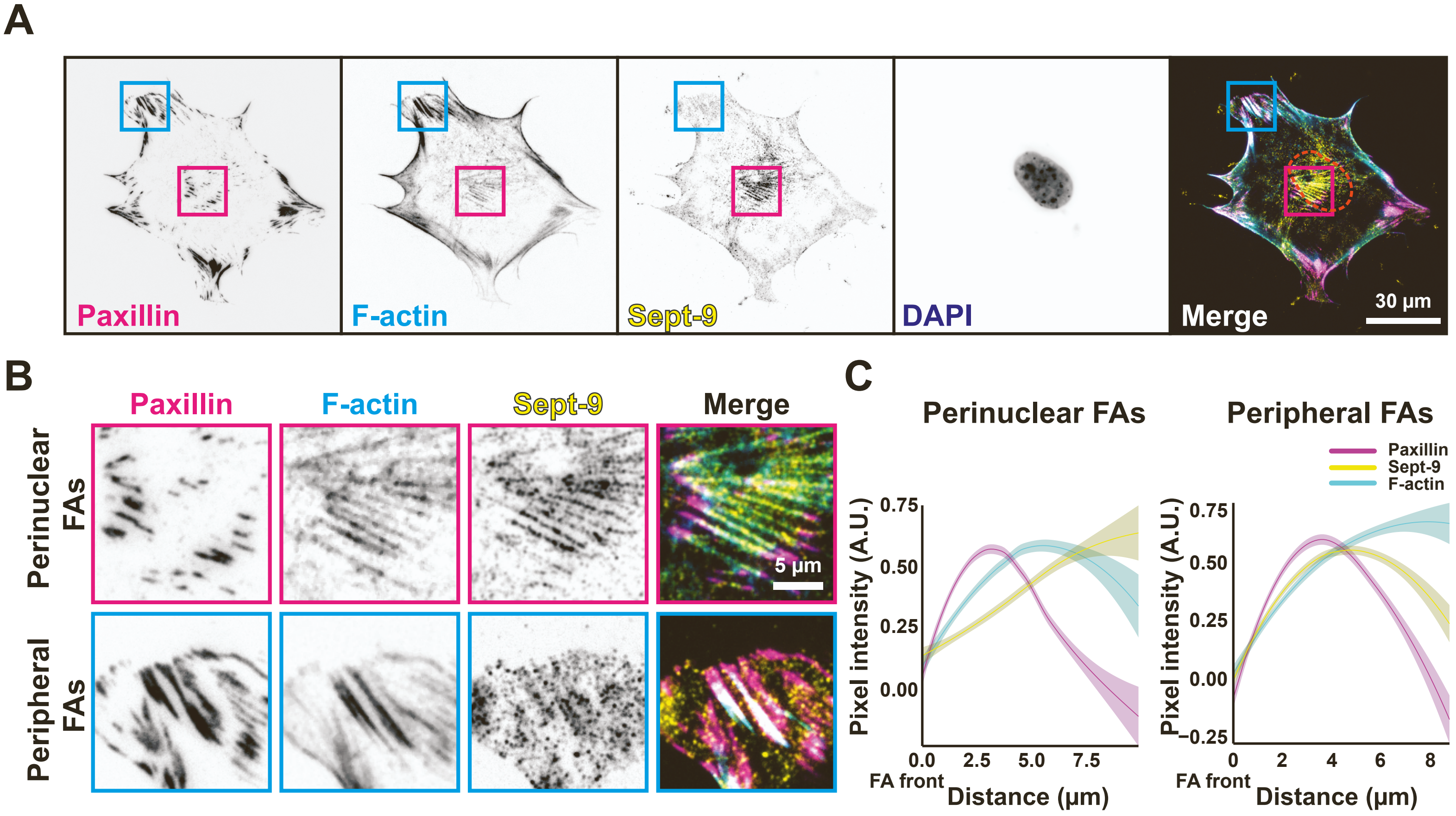

Figure S2. **Localization of Sept-9 at paxillin decorated perinuclear and peripheral FAs, related to figure 1.** (A) Representative TIRFM images of MEFs plated on 10 µg/ml FN stained with paxillin (magenta), F-actin (cyan), and Sept-9 (yellow) and DAPI, magenta and cyan boxes highlight perinuclear and peripheral FAs respectively; scale bar, 30 µm. (B) Insets of perinuclear (magenta), and peripheral (cyan) FAs from (A); scale bar, 5 µm. (C) Curve plots showing normalized line scan intensities of paxillin (magenta), Sept-9 (yellow), and F-actin (cyan), for perinuclear (left) and peripheral (right) FAs, shaded curves represent SD, n = 2 replicates, 23 – 24 line scans.

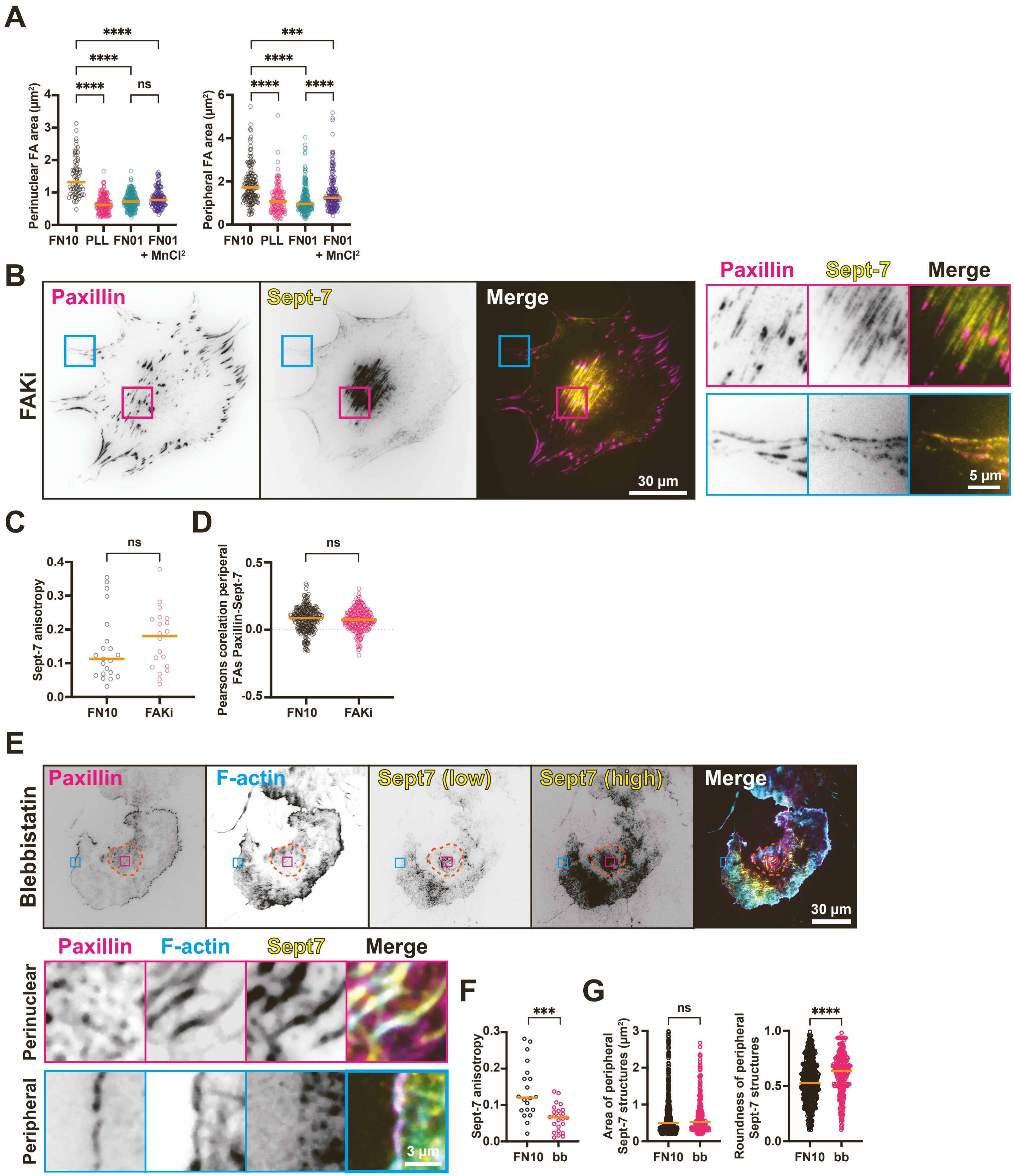
A
Perinuclear FA area (µm²)
Peripheral FA area (µm²)
FN10 PLL FN01 FN01 + MnCl²
ns
B
FAKi
Paxillin
Sept-7
Merge
30 µm
5 µm
C
Sept-7 anisotropy
FN10 FAKi
D
Pearsons corelation periperal FAs Paxillin-Sept-7
FN10 FAKi
E
Blebbistatin
Paxillin
F-actin
Sept7 (low)
Sept7 (high)
Merge
30 µm
Sept7
Perinuclear
Peripheral
3 µm
F
Sept-7 anisotropy
FN10 bb
G
Area of peripheral Sept-7 structures (µm²)
Roundness of peripheral Sept-7 structures
FN10 bb

Figure S3. **FA properties, and perinuclear and peripheral Sept-7 properties in response to inhibition of FAK or myosin activity, related to figure 2.**
(A) Quantification of perinuclear and peripheral FA area of MEFs plated on 10 µg/ml FN, PLL, 0.1 µg/ml FN, and 0.1 µg/ml FN + $MnCl^2$, n = 3 replicates, 750 - 2310 FAs. (B) Representative TIRFM images of MEFs plated on 10 µg/ml FN and treated for 2 hr with focal adhesion kinase (FAK) inhibitor and stained for paxillin (magenta), and Sept-7 (yellow). Magenta and cyan boxes indicate perinuclear and peripheral FAs respectively with insets shown on the right; scale bar, 30 µm (main images), 5 µm (insets). (C) Quantification of Sept-7 anisotropy of MEFs plated on 10 µg/ml FN with and without FAK inhibitor, n = 2 replicates, 20 - 21 cells. (D) Quantification of colocalization between paxillin and Sept-7 at peripheral FAs of cells plated on 10 µg/ml FN with and without FAK inhibitor, n = 2 replicates, 2280 – 2540 FAs. (E) Representative TIRFM images of MEFs plated on 10 µg/ml FN and treated for 2 hrs with blebbistatin and stained for paxillin (magenta), F-actin (cyan), and Sept-7 (yellow, low and high resolution). Magenta and cyan boxes indicate perinuclear and peripheral FA insets respectively (lower panels); scale bar, 30 µm (main images), 3 µm (insets). (F) Quantification of Sept-7 anisotropy of MEFs plated on 10 µg/ml FN, and 10 µg/ml FN with blebbistatin, n = 2 - 3 replicates, 20 – 24 cells. (G) Quantification of peripheral Sept-7 structure area (left plot), and roundness (right plot), for MEFs plated on 10 µg/ml FN, and 10 µg/ml FN with blebbistatin, n = 2 replicates, 431 – 532 structures. All statistics performed using a Mann-Whitney test or Kruskal-Wallis with Dunns multiple comparisons **** $P < 0.0001$, *** $P < 0.001$, ** $P < 0.01$, * $P < 0.05$, ns, not significant, orange horizonal lines show medians.

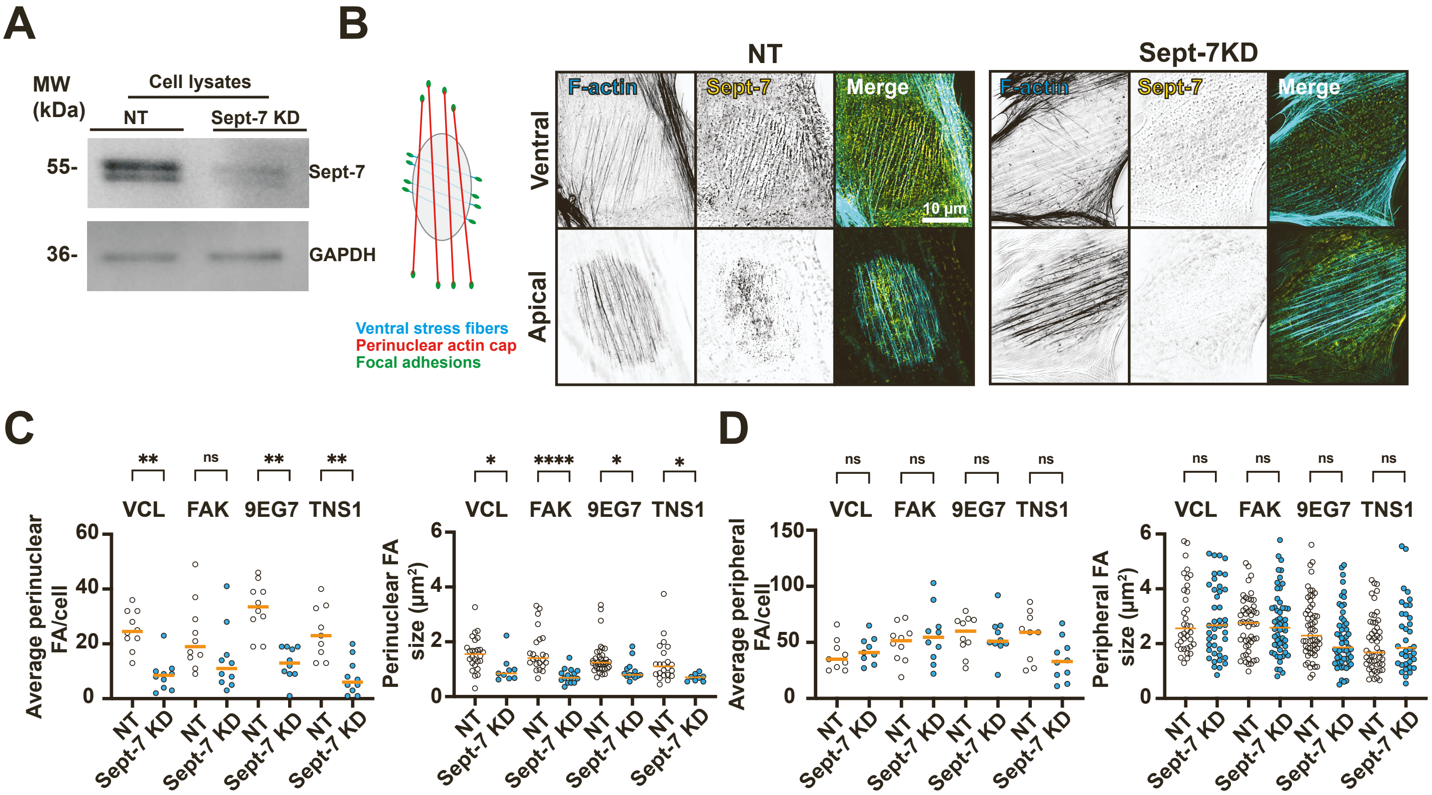

Figure S4. **Effects of Sept-7 knockdown on F-actin, and perinuclear and peripheral adhesions stained with various adhesion proteins, related to figure 3.**

(A) Representative Western Blotting images of cell lysates from MEFs showing Sept-7 and GAPDH after transfecting and incubating cells for 48 hours with NT or Sept-7 siRNA. (B) Representative 3D Structured Illumination Microscopy (SIM) images showing ventral SFs (ventral) and perinuclear actin cap (apical) of MEFs plated on 10 µg/ml FN treated with NT or Sept-7 siRNA and stained for F-actin (cyan), and Sept-7 (yellow); scale bar, 10 µm, cartoon depicts the perinuclear actin cap apical to the nucleus, ventral SFs ventral to the nucleus, and FAs, of MEFs. (C) Quantification of the average perinuclear FAs per cell (left plot), n = 1 experiment 9 -10 cells, and FA size (right plot), n = 1 experiment, 94 - 332 FAs of MEFs treated with NT or Sept-7 siRNA and probed for FA associated proteins vinculin (VCL), focal adhesion kinase (FAK), active a5b1 integrin (9EG7), and tensin-1 (TNS1). (D) Quantification of the average peripheral FAs per cell (left plot), n = 1 experiment, 9 -10 cells, and FA size (right plot), n = 1 experiment, 360 - 580 FAs of MEFs treated with NT or Sept-7 siRNA and probed for FA associated proteins vinculin (VCL), focal adhesion kinase (FAK), active a5b1 integrin (9EG7), and tensin-1 (TNS1). All statistics performed with Kruskal-Wallis and Dunns multiple comparisons test, **** P < 0.0001, *** P < 0.001, ** P < 0.01, * P < 0.05, ns, not significant, orange horizonal lines show medians.

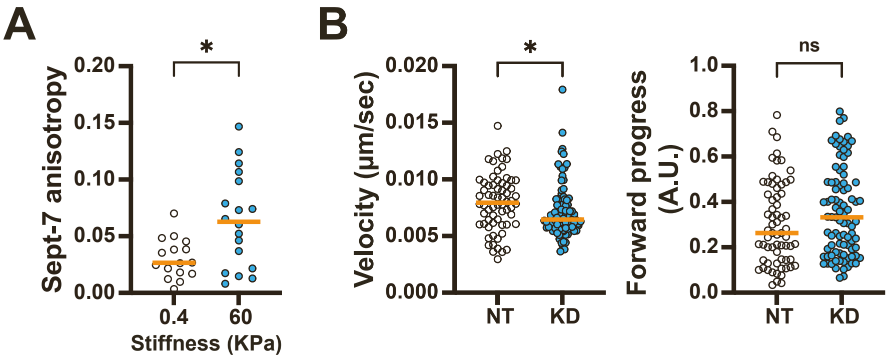

Figure S5. **Cell migration properties of Sept-7 knockdown cells, relating to figure 4.**
(A) Quantification of Sept-7 anisotropy of MEFs plated on soft or stiff polyacrylamide gels, n = 16 - 18 cells. (B) Quantification of cell velocity and forward progress of NT and Sept-7 KD MEFs imaged over 12 hr, n = 2 replicates, 71 – 89 cells. All statistics performed using Mann-Whitney test, **** P < 0.0001, *** P < 0.001, ** P < 0.01, * P < 0.05, ns, not significant, orange horizonal lines show medians.
